# Fear Circuits in Panic Disorder: An Update

**DOI:** 10.31083/AP44174

**Published:** 2025-06-24

**Authors:** Peter Kyriakoulis, Rafael Christophe da Rocha Freire

**Affiliations:** ^1^Department of Psychiatry, Positive Psychology Centre, Melbourne, VIC 3931, Australia; ^2^Department of Psychiatry and Centre for Neuroscience Studies, Queen’s University Kingston, Kingston, ON K7L 4X3, Canada

**Keywords:** fear circuitry, panic disorder, anxiety disorder, neurobiology, neuropathology and neurophysiology

## Abstract

**Objectives:**

Findings from animal models have been instrumental in elucidating the mechanisms and etiology of panic disorder (PD); nonetheless, several aspects of its neurobiological underpinnings remain to be fully clarified. This review aims to consolidate current understanding and recent advances in the neuroanatomical and pathophysiological basis of PD.

**Method:**

A narrative review was conducted, drawing on recent literature addressing the neurobiology and neuroanatomy of PD, with a particular focus on fear circuits as elucidated by both preclinical and clinical studies.

**Results:**

This updated review further delineates the fear circuitry implicated in PD, emphasizing the roles of the amygdala, thalamus, hippocampus, insula, and prefrontal cortex in the mediation of pathological fear responses.

**Conclusion:**

Continued research involving human populations is essential to refine current models of fear circuitry in PD. Such efforts may yield critical insights that support the development of evidence-based therapeutic strategies aimed at re-establishing disrupted homeostatic processes that have been disrupted by the activation of the brain’s fear circuitry.

## Main Points

- Panic disorder is a prevalent anxiety disorder which is associated with distress, disability and poor quality of life.
- A number of interconnected brain structures have been implicated in panic disorder, including amygdala, thalamus, hippocampus, insula, locus coeruleus, periaqueductal gray matter, anterior cingulate cortex and bed nucleus of the stria terminalis.
- Risk assessment and fear conditioning in PD seems to be mediated by the hippocampus.
- Serotonergic, GABAergic and opioidergic systems are fundamental in the neurobiology of PD.

## 1. Introduction

Panic Disorder (PD) is a complex and severe anxiety disorder characterized by heightened distress that can often be occupationally, personally and socially disabling [1, 2]. The 12-month prevalence of anxiety disorders ranges from 14.0% to 19.9% in the general population [3, 4] and these disorders are the most important causes of disability and work impairment, along with chronic pain and mood disorders [5, 6, 7]. Although the lifetime prevalence of panic disorder (4.7%) is lower compared to other anxiety disorders, it is still noteworthy [8]. PD has resulted in significant levels of interpersonal, occupational, and physical disability [1], making it one of the most costly mental health conditions in primary healthcare and community settings [8, 9, 10]. The lifetime prevalence of subthreshold PD and panic attacks (PAs) is notably high, at 22.7% [11] and is associated with high individual and social costs [12].

When an individual perceives a stimulus as potentially threatening, a series of adaptive responses involving neurochemical, neuroendocrine, and behavioral changes are triggered to enhance the chances of survival. These neurobiological fear responses form the fear circuitry, which includes key brain regions such as the amygdala, thalamus, hippocampus, insula, and prefrontal cortex [13].

Several neuroanatomical models have been proposed to elucidate panic and investigate the brain’s fear circuits involved in the disorder [14, 15, 16, 17]. Animal studies have been instrumental in understanding the mechanisms and etiology of panic disorder, significantly contributing to our current knowledge of the brain’s fear circuitry, however, much has yet to be determined [18]. With the advancement of research on PD over time, it is necessary to aggregate important information about the debilitating disease. Based on this, this study aimed to conduct a literature review, considering the main studies and findings on the fear circuitry in PD.

In this review, we will cover the following theories and brain regions implicated in the fear circuitry involved in PD: (1) the neuroanatomical theory of PD (2) the central nucleus of the amygdala (3) the role of the hippocampus (4) the circa strike defence (5) dorsal raphe nucleus (6) Deakin and Graeff hypothesis (7) the role of Gamma-aminobutyric acid (GABA) and (8) the role of opioids in PD. Moreover, the explanations of brain structures, and a comparison of theories are discussed in order to explore what we know and don’t know in the area of fear circuitry and PD, addressing the limitations regarding the neuroanatomical and neurochemical mechanisms underlying PD. Finally, we will discuss anticipated future developments in this area.

## 2. Neuroanatomical Theory of PD

The neuroanatomical theory of PD, first proposed by Gorman and colleagues, suggests that dysfunctional integration of information and lack of coordination between cortical and subcortical neural circuitry contribute to PD [14]. Gorman hypothesized that PAs stem from increased activity in noradrenergic neurons of the locus coeruleus (LC), which is responsible for panic and stress responses. Whilst anticipatory anxiety is linked to limbic structures, the prefrontal cortex (PFC) is thought to influence phobic avoidance [18].

Gorman’s team proposed that treatments like selective serotonin reuptake inhibitors (SSRIs) may alleviate PAs by reducing amygdala activity and inhibiting projections to subcortical areas, including the brainstem. They also suggested that cognitive-behavioral therapy (CBT) may help by deconditioning contextual fear and enhancing the PFC’s ability to inhibit the amygdala [14].

In Gorman’s revised hypothesis, neuroanatomical pathways in humans were mapped, highlighting parallels between conditioned fear responses in animals and PAs in humans [15]. Despite its popularity, the model faced criticism, particularly from studies showing that Urbach–Wiethe disease patients, who lack an amygdala, can still experience PAs [19]. Additionally, the noradrenergic theory of PD has been challenged, with some researchers arguing that the LC primarily mediates arousal rather than panic.

## 3. Central Nucleus of the Amygdala 

Recent insights into brain pathways and neurocircuitry have allowed for the development of conditioned fear responses and the fear network. According to LeDoux [20] the amygdala has been the central hub of fear processing networks and is closely associated with the pathogenesis of PD as well as PAs. Amongst the fear structures playing a central role in panic, is the central nucleus of the amygdala (CeA), where it is considered that PAs originate [21]. It is closely connected to the thalamus which serves as a crucial relay station for sensory information, processing inputs from all senses. It analyzes external threats and evaluates bodily signals that may indicate danger.

It initiates two strategies in response to perceived threats. The first one is an emergency response, known as the downstream pathway which involves the nucleus of the solitary tract via the parabrachial nucleus or the sensory thalamus. The second strategy is a more gradual and detailed analysis of the threatening situation, which involves an upstream response, allowing for a higher-level neurocognitive processing and the nuanced interpretation of sensory information [16, 22].

The viscerosensory input for the fear stimulus is processed via the thalamus to the lateral nucleus of the amygdala and conveyed to the CeA. Here, all gathered information is integrated, allowing for the organization and dissemination of autonomic and behavioral responses.

**Fig. 1.**
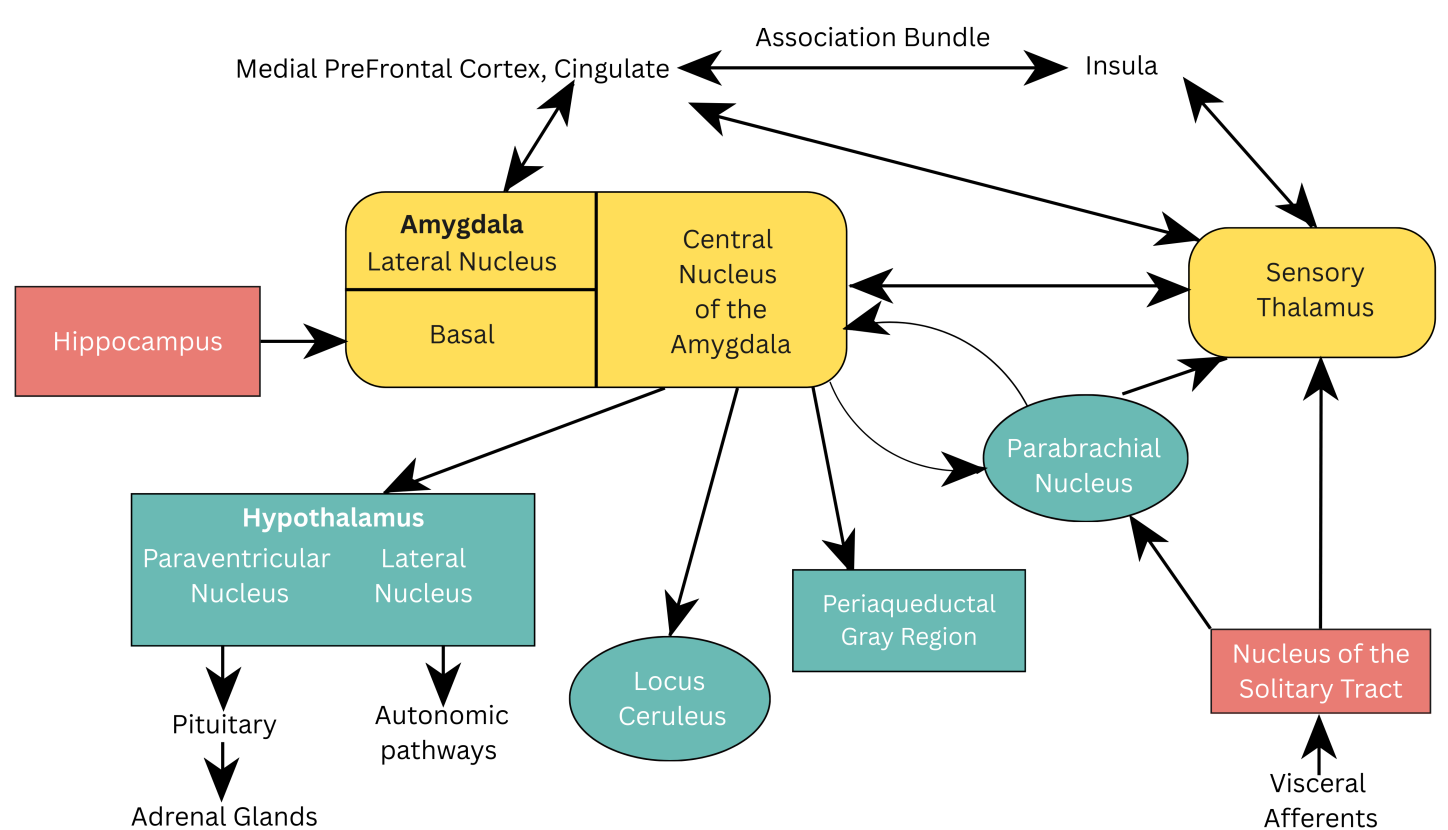
**Neuroanatomical pathways of viscerosensory information in the brain**. Reproduced with permission from Jeremy D. Coplan, Neuroanatomical hypothesis of panic disorder, revised; published by The American Journal of Psychiatry, 2000 [16].

The CeA is responsible for sending stimuli to various brain regions, including the parabrachial nucleus, to increase respiration rate, to the lateral nucleus of the hypothalamus, activating the sympathetic nervous system (SNS) and to the paraventricular nucleus of the hypothalamus increasing the release of adrenocorticoids [23]. Furthermore, projections to the LC exaggerate the fear response by producing norepinephrine release and elevations in heart rate and blood pressure. See Fig. 1 (Ref. [16]) which illustrates the neuroanatomical pathways of viscerosensory information in the brain.

Phobias have been associated with both conditioned fears which are learned and innate fears which are inborn and based on threats that are specific to a species. A cardinal brain site for the organisation of innate fears that has been identified is the periaqueductal gray (PAG) [19]. The PAG region receives projections from the CeA which leads to defensive behaviors including avoidance and postural freezing, similar to those experienced by animals when threatened. Freezing is a specific response to a threat, in which all movement ceases except for breathing in an attempt to ensure survival [24]. Freezing can be conceptualized as a parasympathetic brake on the motor system, rather than a passive state, relevant to enhanced perception and action preparation [25]. The freeze response may be the result of the threat that is coming from within rather than our external environment. Innate defence behaviour can also be activated by threatening interoceptive stimuli like hypoxia (lack of oxygen) or hypercapnia (increases in CO₂) [26]. Among the interoceptive stimuli that seem to be crucial for panic patients is the threat of asphyxiation. The insula and the anterior cingulate gyrus (ACG) are the critical brain structures responsible for the detection of internal threats. The central structure that mediates the bidirectional transition between defensive behaviour and the default exploratory behaviour is the amygdala [27, 28]. The basolateral amygdala (BLA) activates the defence circuit via the threatening information that is received by the sensory systems [29]. These defensive circuits prompt autonomic, endocrine and motor responses to counter, proximal and distal threats [28].

## 4. Hippocampus in PD

The hippocampus is responsible for vital cognitive abilities that include spatial recognition and orientation, declarative memory and the regulation of anxiety and mood [13, 30, 31, 32, 33]. The hippocampus contributes to the integration of defensive neural networks that makes up the fear circuitry comprising of the hippocampus, amygdala, nucleus accumbens, periaqueductal gray, ventromedial hypothalamus, thalamic nuclei, insular cortex, several brain stem and prefrontal regions [13]. Along with cortical and subcortical areas, the formation of the hippocampal plays a vital role in the emotional system of the brain and in the modulation of complex behavioural patterns [13].

One of its functions is in the processing of risk assessment which is a fundamental aspect of emotional regulation aimed at appraising potential danger versus rewards [13, 34]. Another role of the hippocampus in PD is in mediating contextual fear learning and the expression of fear and anxiety elicited by learned fear. Research has demonstrated that fear conditioning is interrupted in cases of hippocampus lesions [35], hence one’s ability to predict aversive events may be impaired.

**Fig. 2.**
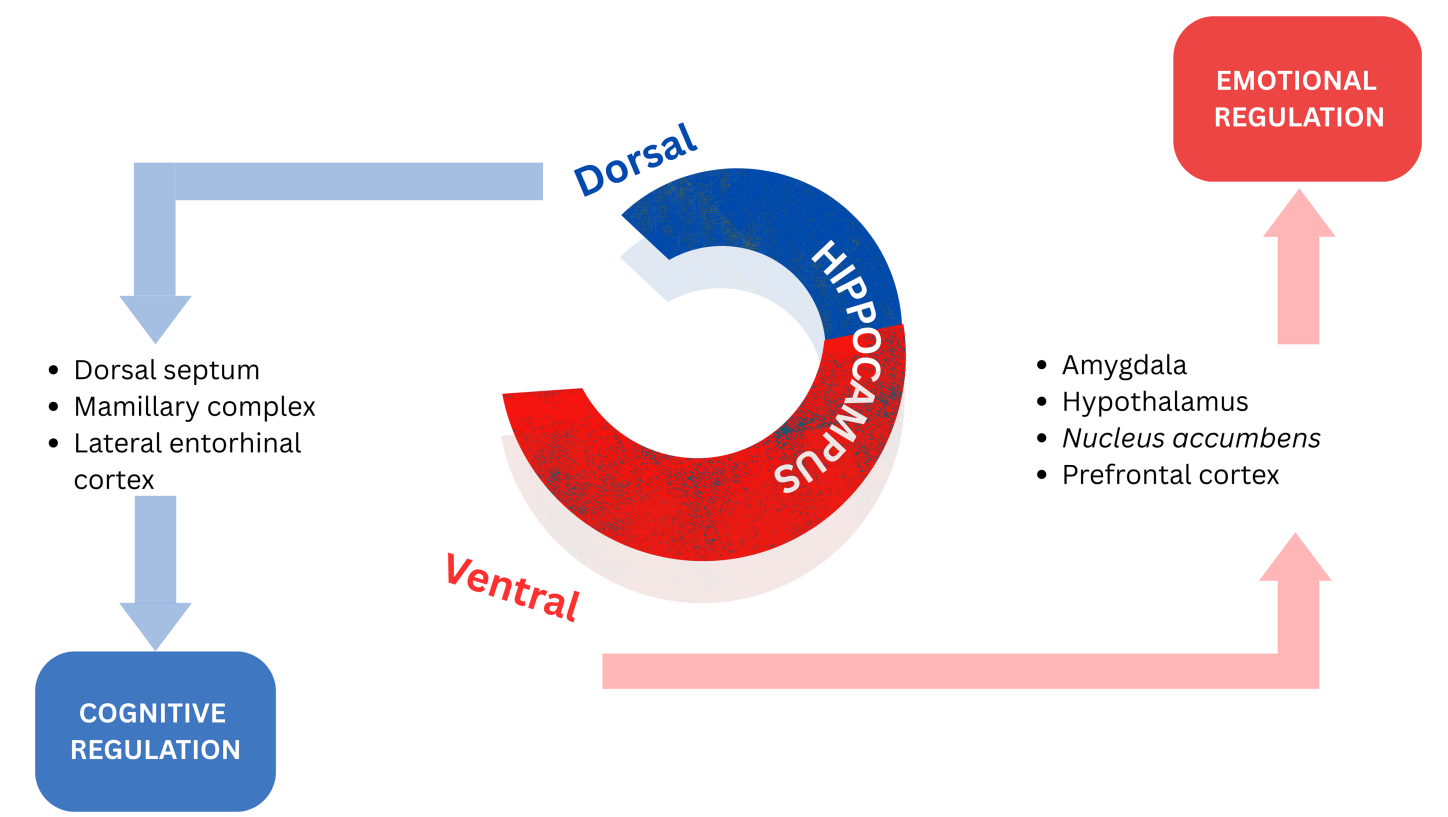
**Schematic representation of the dorsoventral division of the hippocampus**. Reproduced with permission from Sandrine Thuret, The Hippocampus and Panic Disorder: Evidence from Animal and Human Studies; published by Springer Nature, 2016 [13].

Evidence indicates that the hippocampus regulates its functions in a topographically organized manner along its septo-temporal axis. The ventral hippocampus’ projections to limbic areas point to its regulation of emotional processing and gathering salient environmental information from externally and from an individual’s internal physiological state to assist with the consolidation of fear memories. The dorsal hippocampus is more related to the role of cognition, processing information and transforming that into orienting and locomotor actions [13, 36, 37, 38, 39, 40]. Fig. 2 (Ref. [13]) depicts a schematic representation of the dorsoventral division of the hippocampus.

While the role of the hippocampus in emotional regulation has been well demonstrated, further investigations are necessary to determine the specific functional properties and contributions of the dorsal and ventral portions of the hippocampus to the process of emotional regulation [13].

## 5. Circa Strike Defence

The circa strike defence is activated by an imminent threat and is characterized by prominent autonomic arousal and escape behavior that includes active defensive behaviors (e.g., active avoidance, fight, flight) [11]. During the circa strike defence brain regions cooperate to manage or “defend” against inappropriate or excessive fear, and an individual’s behavior under threat transitions from passive freezing to active flight, or even attack if escape is not possible [41]. The dorsal periaqueductal gray (DPAG) mediates the circa strike defence, evoking electrical and chemical stimulations of the DPAG and directing the expression of escape behaviors which is followed by the discharge of the SNS [26, 27, 42, 43, 44, 45]. PAs can be conceptualised as involving the circa strike defence being activated by unconditioned internal physiological threats (e.g., suffocation alarm) which are possibly mediated by the DPAG. This is in line with the learning perspective on the aetiology of PD that was suggested by Bouton and colleagues [46] that claims, strong autonomic arousal and severe fear experienced associated with the first PA can be classified as unconditional circa strike defence activation [28].

Anxious apprehension develops following the experience of severe PAs as conditioned responses to mild body symptoms. Post-encounter defence results from conditioned fear responses and involves increased selective attention and threat appraisal, defensive freezing and startle potentiation [28]. Avoidance behaviors that lead to agoraphobia are understood to be motivated by the survival instinct to remain in a safe context that the individual can control. Additionally, the bed nucleus of the stria terminalis (BNST), a structure in the brain, may play a significant role in the pathogenesis of PD, as it is associated with anxiety responses related to threat monitoring [47, 48]. Walker *et al*. [49] proposed that the BNST is particularly involved in sustained fear which has been conceptualized as playing a role in the contribution of agoraphobia.

**Fig. 3.**
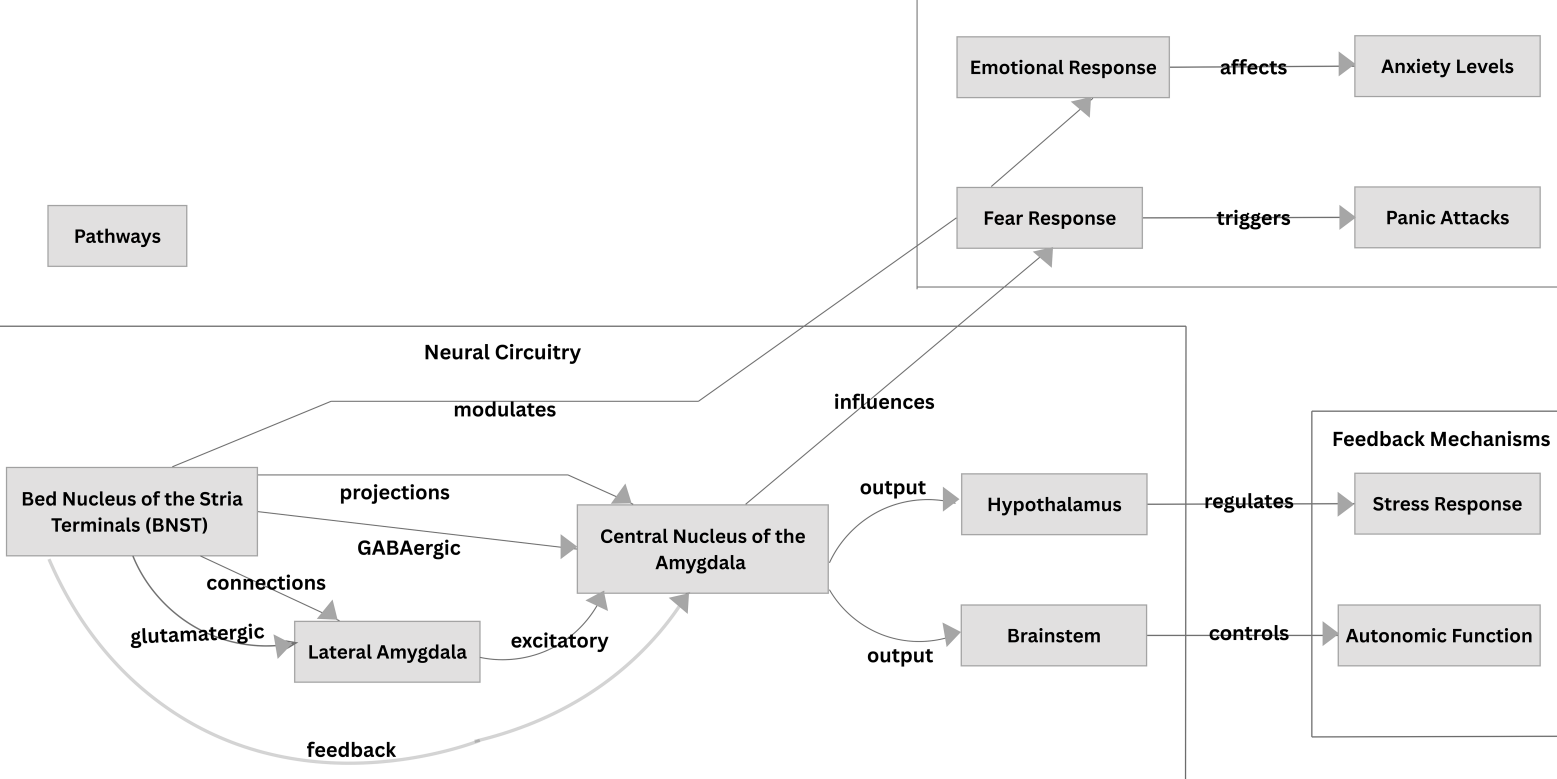
**The Bed Nucleus of the Stria Terminalis and the Amygdala and their projections in the fear circuitry implicated in Panic Disorder and Anxiety**.

A schematic representation of the BNST and the amygdala with its afferent projections to the fear circuitry implicated in PD and anxiety is illustrated in Fig. 3.

## 6. Dorsal Raphe Nucleus in PD

Another neuroanatomical area implicated in PD is the dorsal raphe nucleus (DRN). The subdivisions of the DRN consist of distinct populations of neurons that vary in terms of structure, neurochemistry, and function. Research has shown that various subsets of neurons within the DRN are activated in response to anxiety and panic, respectively [18, 48, 49]. Specifically, anxiety processing is facilitated by serotonin (5-HT) neurons located in the caudal and mid-caudal regions of the DRN, while the lateral wings and adjacent ventrolateral periaqueductal gray (vlPAG) are particularly sensitive to panic-evoking stimuli and conditions [18, 49]. Furthermore, the 5-HT neurons in the DRN wings, along with those in the adjacent vlPAG, typically regulate behavioral and neurovegetative responses to non-threatening interoceptive and exteroceptive stimuli. The dysfunction of this system leads to increased vulnerability to panic responses including PAs in humans [18]. Hypothalamic sites that include the dorsomedial and ventrolateral parts of the ventromedial nucleus and the dorsomedial nucleus, process threats (e.g., social, interoceptive, predator) and relay information to the DRN wings and neighbouring vlPAG [18, 50, 51]. The DRN 5-HT efferents facilitate avoidance during threat anticipation at the amygdala, which at the proximal defence system at DPAG also restrains the fight/flight components [52]. 5-HT and dopamine receptors in the ventral striatum mediate avoidance and approach behaviors, respectively, with conflicting behaviors being processed through these receptors [52].

Deakin and Graeff [53] proposed that Generalized Anxiety Disorder (GAD) is produced by the overactivity of serotonergic 5-HT excitatory projections from DRN to the PFC regions and BLA, which process distal threat, whereas PAs have been conceptualized as being the result of dysfunction of 5-HT inhibitory projections to dorsal regions of DPAG that process proximal threat, innate fear, or hypoxia.

An impaired interaction between serotonin and opioid receptors in the DPAG has been linked to the onset of PAs [52]. Underactivity in the PFC leads to decreases in amygdala inhibition which has been associated with disorders that produce intense fear such as PD, social anxiety, and post traumatic stress disorder (PTSD). Overactivity of PFC has been linked to disorders such as obsessive compulsive disorder (OCD) and GAD, that involve rumination and obsessiveness [53, 54, 55].

## 7. Deakin And Graeff Hypothesis

Deakin and Graeff [51] also suggested that anticipatory anxiety to distal threats including olfactory cues and stimuli was the result of ‘basolateral defence system’ activation that encompasses the PFC and amygdala. Panic-like responses to distal threats that include freezing and directed flight are mediated by the basolateral defence system and the hypothalamus, while panic-like responses to proximal threat that include explosive flight responses are facilitated by the DPAG [52].

Deakin and Graeff [53] established that dysfunction of the ‘rostral defence system’ that includes the medial PFC, amygdala and hypothalamus is responsible for fear, phobias and situational PAs, while faulty ‘caudal defence system’ of the DPAG is responsible for spontaneous PAs [52].

Research has demonstrated that the amygdala is essential for the achievement of fear-potentiated startle but does not have any part in the retention or expression of this response [52, 56, 57]. According to the Deakin-Graeff hypothesis (DGH), in response to a proximal threat, 5-HT efferents of the DRN, inhibit the circuits of DPAG facilitating the fight/flight responses, while during anticipation of threat, it assists by facilitating the PFC and amygdala circuits in mediating avoidance responses [52, 53]. The amygdala has also been linked to aversive learning with research showing that the nuclei of the dorsal amygdala play a vital role in the development of conditioned fear to tone or light that was earlier paired to a shock [52, 58, 59, 60, 61, 62, 63, 64].

Research using the combination of operant conditioning and brain stimulation has led to the discovery of aversive and reward systems in the brain. Specifically, research conducted by [65] demonstrated that the stimulation of the lateral hypothalamus led to a strong approach response, while the opposite occurred with the stimulation of the periventricular hypothalamus and the PAG which resulted in only negative reinforcement [65, 66, 67]. In summary, while the Deakin-Graeff hypothesis emphasizes serotonin’s role in regulating anxiety, Gorman’s theory focuses on the brain circuits involved in fear processing. Together, they complement each other and provide a more comprehensive understanding of anxiety [68].

## 8. GABA in PD

GABA is synthesized and released throughout the brain, functioning as one of the major inhibitory neurotransmitters that dampen activity in panic-generating regions such as the perifornical hypothalamus (PeF) and the DPAG. There is a strong association between GABA and PAs as well as PD, with studies indicating that individuals with PD exhibit deficits in GABA activity [43]. Pharmacological interventions have been shown to restore GABA activity, highlighting its importance in managing panic symptoms. Additionally, panic vulnerability may disrupt GABA inhibition in the dorsomedial/perifornical hypothalamic (DMH/PeF) region, which mediates the respiratory and autonomic components of the panic response. This disruption is reflected in increased autonomic arousal and the elicitation of anxiety and freezing responses in fearful situations [69, 70, 71, 72]. Research has demonstrated that PD patients demonstrated reduced binding of Gamma-aminobutyric acid Type A (GABAA) receptors in the frontal cortex [73], and a study by Goddard and colleagues [74] found deficits in central GABA concentrations among PD patients.

## 9. Opioids in PD

Various neurochemical theories have been proposed to explain the onset of PD, including the possibility of either a deficiency or an excess of serotonin in the system [54], both of which could contribute to vulnerability as well as potential adaptive responses. Johnson and colleagues [41] argued that vulnerability to PAs may result from impaired serotonergic inhibition of the DPAG and the autonomic medullary centres.

Some candidate genes including the serotonin transporter gene, and the serotonin 2A receptor gene, have been found in serotonergic and non-adrenergic systems which are associated with PD and the severity of PAs [68, 72]. Moreover, Preter and Klein [75] proposed that dysfunction in the endogenous opioid system lowers the threshold for the suffocation alarm. This hypothesis was supported by Preter and colleagues [76], who found that lactate infusions in naloxone-treated healthy participants induced feelings and symptoms closely resembling those of PAs. Additionally, other neurochemical theories have identified a dense concentration of neuropeptide Y in anxiety circuits, suggesting its involvement in the consolidation of fear memories [77].

Research by Preter and Klein [78] conclusively established a physiological link between panic-like suffocation in healthy adults and a deficiency in the endogenous opioid system. They argued that episodic dysfunction of the opioidergic systems leads to a decreased threshold for the suffocation alarm, resulting in the occurrence of PAs [52, 78]. Furthermore, Graeff [54] proposed that endorphins heighten sensitivity to suffocation and exacerbate separation anxiety in individuals with PD, thereby increasing their vulnerability to experiencing PAs.

The internal homeostatic environment, which involves the regulation of parameters like pH balance and chemosensation, is a critical area of study that deepens our understanding of panic pathophysiology and treatment. It is hypothesized that PAs may arise from dysfunction in 5-HT inhibitory projections to the DPAG, which processes proximal threats, innate fear, or hypoxia. Additionally, a faulty interaction between 5-HT and opioid receptors in the DPAG has been linked to the onset of PAs [54].

Table 1 presents the key findings of this narrative review.

**Table 1.** **Key findings of the fear circuitry in panic disorder**.

Category	Key findings
Key brain structures	- Amygdala: Central to fear processing; particularly the Central Nucleus of the Amygdala (CeA) is involved in panic responses.
	- Prefrontal Cortex (PFC): Influences inhibition of the amygdala and regulates fear and anxiety.
	- Hippocampus: Important for contextual fear learning and emotional regulation; impairments in the hippocampus impacts risk assessment.
	- Locus Coeruleus (LC): Associated with panic and stress responses; norepinephrine release linked to panic attacks.
	- Dorsal Raphe Nucleus (DRN): Contains serotonergic neurons; dysfunction increases vulnerability to panic responses.
Neurochemical systems	- Gamma-aminobutyric acid (GABA): Major inhibitory neurotransmitter; deficiency linked to heightened anxiety and panic.
	- Serotonin: Modulates anxiety and panic; dysfunctional pathways affect responses to threats.
	- Endogenous Opioid System: Dysfunction may lower the threshold for panic responses, especially to suffocation.
Fear circuits	- Conditioned and Innate Fears: Fear responses can be learned or inherent; specific circuits regulate each type.
	- Circa Strike Defense Mechanism: Panic attacks activate this defense system, leading to autonomic arousal and escape behaviors.
Behavioral responses	- Individuals with panic disorder (PD) exhibit heightened responses to perceived threats, leading to increased anxiety and avoidance behaviors.

## 10. Discussion

In summary, the brain’s fear circuitry involves several key regions, including the amygdala, prefrontal cortex (PFC), hippocampus, insula, thalamus, dorsal raphe nucleus (DRN), and locus coeruleus (LC) [13]. The thalamus relays sensory input to both the amygdala and cortex for processing. The amygdala, particularly the CeA, detects interoceptive and exteroceptive threats, triggering fear responses, while the PFC modulates these reactions. The hippocampus provides context, and the insula processes bodily fear responses. In PD, dysregulation of neural circuits contributes to heightened fear responses. Serotonin, primarily originating from the DRN, plays a key role in modulating the amygdala and prefrontal cortex (PFC) to regulate emotional processing [79]. Impaired serotonin function increases fear sensitivity, which is believed to contribute to the occurrence of PAs [80].

The LC, which releases norepinephrine, enhances alertness and arousal and interacts with the amygdala and PFC to amplify fear responses while GABAergic dysfunction and opioid systems further impair fear regulation. The concept of circa-strike defence involves rapid, adaptive fear responses; however, in PD, this system becomes maladaptive, leading to exaggerated fear and impaired regulation. Collectively, these brain regions and systems form a complex network that governs both immediate and regulated fear responses [81].

The Deakin-Graeff hypothesis is a neurochemical theory that emphasizes the role of serotonin (5-HT) and its interaction with specific receptors (especially 5-HT1A and 5-HT2A/2C) in regulating anxiety, suggesting that serotonergic dysfunction contributes to PD [53]. Serotonin plays a central role in anxiety regulation, supporting treatments such as SSRIs that target serotonergic systems. In contrast, Gorman’s neuroanatomical theory focuses on the brain circuits underlying anxiety, particularly the interaction between the amygdala, involved in fear processing, and the prefrontal cortex (PFC), which regulates emotional responses. Gorman proposes that dysfunction within these circuits, along with alterations in the hippocampus and brainstem, contributes to exaggerated fear responses. While Deakin-Graeff highlights serotonin as a key player, Gorman focuses on brain circuitry, especially the amygdala-PFC network. These theories are complementary, as neurochemical imbalances like serotonin dysfunction and brain circuit dysfunction contribute to PD, pointing to the need for treatments that target both neurotransmitter systems and brain circuitry. The Deakin-Graeff hypothesis supports serotonin-based treatments (e.g., SSRIs), while Gorman advocates for approaches that target brain circuit regulation, such as cognitive therapies and neuromodulation [16].

Recent research has elucidated the neuroanatomical and neurochemical mechanisms underlying PD and PAs emphasizing the central role of the central nucleus of the amygdala (CeA) in fear processing [21]. The amygdala integrates sensory information relayed by the thalamus and coordinates autonomic and behavioral responses to perceived threats. This integration facilitates both immediate emergency responses and nuanced cognitive evaluations of danger.

The periaqueductal gray (PAG) is crucial for innate fear responses, contributing to the freezing response, which is an active survival strategy rather than a passive state. Additionally, the hippocampus plays a key role in emotional regulation and contextual fear learning, with distinct functions attributed to its ventral and dorsal regions [37]. However, further research is needed to clarify these specific contributions.

Neurochemical theories highlight the roles of the serotonergic and opioid systems in PD, with serotonergic dysfunction linked to impaired inhibition of panic-related pathways. This suggests that PAs may arise from a complex interplay of neurochemical imbalances. The circa strike defence model illustrates how the DPAG mediates responses to internal threats, reinforcing the significance of autonomic arousal [50]. The DRN also plays a pivotal role in panic vulnerability, as distinct neuron populations within the DRN respond variably to anxiety and panic [79, 80]. Moreover, the involvement of GABA as a major inhibitory neurotransmitter underscores its importance in regulating panic responses, with research indicating GABA deficits in panic patients [43].

While these findings provide valuable insights into the neurobiology of PD, the complexity of panic responses calls for more integrative studies. Neuroimaging studies have highlighted the crucial role of the CeA in responding to threat-related stimuli. The CeA and the BNST share similar connectivity, cellular features, and neurochemistry, both being particularly sensitive to uncertain or distant threats [82]. These studies provide *in-vivo* evidence, consistently activating key brain regions involved in aversive conditioning and extinction, including the amygdala, anterior cingulate cortex (ACC), and insular cortex. This activation pattern aligns with earlier animal studies, supporting the roles of these regions in emotional learning. Research on conditioned fear emphasizes the amygdala’s central role, with the lateral amygdala involved in fear acquisition and the central nucleus in fear behaviors [82].

Human neuroimaging, primarily using functional magnetic resonance imaging (fMRI), has been instrumental in identifying the neural circuits underlying fear conditioning and extinction. By measuring blood oxygen-level dependent (BOLD) signals, fMRI has pinpointed brain regions like the amygdala, ACC, insula, hippocampus, and vmPFC, which are involved in emotional learning. Advances in functional connectivity analysis have further clarified how these regions communicate during fear-related tasks. Other techniques, such as positron emission tomography (PET), electroencephalography (EEG), and magnetoencephalography (MEG), have been used to examine metabolic and electrical activity changes [83]. Particularly PET studies have discovered neurotransmitter imbalances in PD [84]. These studies not only confirm the involvement of regions identified in animal models but also deepen our understanding of the neural mechanisms of emotional learning. Additionally, they are beginning to uncover the biological systems—genes, neurotransmitters, and hormones—that modulate emotional learning and contribute to individual differences in fear conditioning and extinction [82].

Current models often compartmentalize brain structures and neurotransmitter systems, potentially oversimplifying their interactions, hence much remains unknown. Advanced imaging techniques could enhance our understanding of real-time neural dynamics during panic attacks. Significant individual variability in PD symptoms is common, with unclear neurobiological mechanisms. While serotonin and GABA are well-studied, the roles of other neurotransmitters like dopamine and neuropeptide Y need further investigation. Most research is cross-sectional, limiting our understanding of causal relationships, and longitudinal studies are essential for clarity. Additionally, the interplay between neurobiological and psychological factors in shaping fear responses requires exploration. Findings from animal models may not translate well to humans due to emotional complexity [85]. While treatments like SSRIs and CBT are effective, their specific neuroanatomical and neurochemical mechanisms remain underexplored. Lastly, more research is needed on regions of the brain like the insula, anterior cingulate cortex, and periaqueductal gray to fully understand their roles in PD [28].

## 11. Limitations

The theories regarding the neuroanatomical and neurochemical mechanisms underlying PD and PAs provide valuable insights, but they also have several notable limitations. They often oversimplify by isolating brain regions and neurotransmitters, neglecting their complex interactions. Additionally, much research relies on cross-sectional studies, hindering insights into causal relationships, while individual variability complicates generalizability. Many theories emphasize biological factors, overlooking psychological and environmental influences [85]. For instance, the role of GABA dysfunction in panic disorder remains poorly understood, and existing models tend to focus on serotonin and opioids, often ignoring other neurotransmitters like dopamine. Moreover, findings from animal studies may not translate to humans. Animal studies on fear circuitry have been criticised as they often oversimplify fear responses and don’t capture the full complexity of human emotions and cognitions [85]. Moreover, animals lack the social and environmental factors that influence human fear, and real-life experiences like trauma and stress responses are not typically reflected in lab conditions [85]. More notably, treatments that may be effective in animals may not work the same way in humans due to differences in brain and body function [81]. Ethical constraints in manipulating neurochemical systems restrict experimental approaches, underscoring the need for multidimensional models that integrate neuroanatomical, neurochemical, psychological, and environmental factors. Lastly, the wide range of panic symptoms poses challenges for the applicability of existing theories and treatment strategies.

## 12. Conclusion

In conclusion, the multifaceted nature of PD suggests that effective interventions should consider the interplay of neuroanatomical structures, neurochemical systems, and individual psychological contexts to address the disorder comprehensively. In certain cases, combining treatments is preferred for managing panic disorder (PD), highlighting the need for an integrated approach due to the complex and multifactorial nature of its etiology [84]. To better understand panic and the brain’s fear circuits, several neuroanatomical models have been proposed. One such model suggests that hypersensitivity in the brainstem and amygdala plays a role in the pathogenesis of PD [86].

The fear network is activated and includes both subcortical and cortical regions, such as the amygdala, thalamus, hypothalamus, insula cortex, and prefrontal cortex, thereby supporting the validity of neuroanatomical theories [18].

Metabolism and perfusion studies have implicated the hippocampus in the fear circuitry and PD, given its significant role in emotional regulation and in contextualizing fear responses [79]. A few neurotransmitters that have lower receptor binding in the amygdala including GABAA and serotonin, have been reported [87].

Complex emotional and cognitive processing in neuropsychiatric illnesses is associated with abnormal functioning of neural circuits, which incorporate several brain regions, with each neural circuit contributing to different features of a disorder [18]. It has also been demonstrated that different sets of neural circuits are responsible for varying types of defensive responses [49]. Therefore, an understanding of the brain regions involved, and their functional connectivity may further inform our understanding of the neurobiological foundation of PD, further leading to the development of effective interventions incorporating pharmacological, psychotherapeutic/environmental and dietary interventions [18].

Animal studies have played a significant role in informing our understanding of the aetiology, mechanisms, and fear circuitry involved in PD. However, much has yet to be established with regard to the neurobiological basis and pathophysiology of PD. In particular, there is a need for more advanced translational models to identify which animal research has empirical value for humans and to deepen our understanding of the molecular and neural systems involved in panic disorder (PD). Additionally, with recent advancements in neuroimaging technologies, more human research is essential to uncover the underlying mechanisms and gain new insights into the fear circuitry involved in PD. These findings could ultimately inform the development of new treatments for PD sufferers, aimed at restoring homeostatic parameters disrupted by the activation of the brain’s fear circuitry.
